# Evaluating person‐centredness for frail older persons in nursing homes before and after implementing a palliative care intervention

**DOI:** 10.1002/nop2.408

**Published:** 2019-11-19

**Authors:** Christina Bökberg, Lina Behm, Birgitta Wallerstedt, Gerd Ahlström

**Affiliations:** ^1^ Department of Health Sciences Faculty of Medicine Lund University Lund Sweden; ^2^ Department of Health and Caring Sciences Faculty of Health and Life Sciences Centre for Collaborative Palliative Care Linnaeus University Växjö Sweden

**Keywords:** elderly care, nurses, nursing homes, palliative care, person‐centred care, staff education

## Abstract

**Aim:**

To evaluate person‐centeredness in nursing homes from the perspective of frail older persons, before and after implementing an educational intervention about palliative care.

**Design:**

A crossover design.

**Methods:**

Forty‐four older persons living in nursing homes were interviewed. A convergent mixed‐method was used to analyse data.

**Results:**

The older persons expressed feelings of unsafety related to shortcomings in staff. These shortcomings implied that the responsibilities of everyday activities and making the residents’ existence more bearable were transferred to the next of kin. The dropout rate related to death and not enough energy was considerably high (51%) even though one of the inclusion criteria was to have enough energy to manage a 1‐hr interview. This result supports previous research describing the difficulties in retaining older persons in research and indicated that the dose of the intervention was not sufficient to improve person‐centred care.

## 1 INTRODUCTION

It is difficult to imagine anything more personal than death and dying. Although 71% of all people who died in Sweden in 2018 were >75 years (National Board of Health and Welfare, 2019), and 38% of all deaths in Sweden occur in nursing homes (Håkanson, Öhlén, Morin, & Cohen, 2015), older persons dying of multiple progressive morbidities or “old age” at nursing homes have received far less palliative care. This may cause unnecessary suffering and decreased quality of life during the final stage of life (Smedbäck et al., 2017). Furthermore, in Sweden, the medium length of stay after moving into a nursing home is decreasing (Schon, Lagergren, & Karleholt, 2016), and almost one‐third of all the older persons who move into a nursing home die in six weeks (Smedbäck et al., 2017). This situation implies that most of the persons living in nursing homes are in the final stage of life and benefitting from a person‐centred palliative care. Hence, there is an urgent need to implement person‐centred palliative care for older persons in nursing homes (Davies & Higginson, 2004; Froggatt et al., 2017; Smedbäck et al., 2017).

## 2 BACKGROUND

The goals of palliative care are to reduce suffering and promote quality of life for persons with progressive, incurable illnesses or injuries (Connor & Sepulveda Bermedo, 2014; Davies & Higginson, 2004; Hall, et al., 2011). Thus, the care provided needs, among other things, to be person‐centred. The approach in both palliative care and person‐centred care is characterized by a holistic view of the person, and that the person should be supported to live a life with dignity. Person‐centred palliative care strives to make the whole person visible and prioritizes the satisfaction of spiritual, existential, social, psychological and physical needs (Edvardsson, Winblad, & Sandman, 2008; Ekman et al., 2011). Person‐centred care is designed to make nursing and the care environment more personal and to understand behaviours and psychological symptoms from the perspective of the person (Jing, Willis, & Feng, 2016; McCormack, 2003). Although professional caregivers believe that they apply person‐centred care (Ekman et al., 2011), studies have pointed out lack of person‐centred care for older people (Beck, Törnquist, Broström, & Edberg, 2012; McCormack, Karlsson, Dewing, & Lerdal, 2010).

While there is an increasing empirical base for person‐centred care, little research has been undertaken to evaluate the person‐centredness as a result of implementing palliative care in nursing homes (Davies & Higginson, 2004). Therefore, it is essential to ask the person who is the subject of palliative care. The aim of this study was to evaluate person‐centeredness in nursing homes from the perspective of frail older persons, before and after implementing an educational intervention about palliative care. The overall research question in focus in this study is whether the older persons' preferences, wishes and self‐reported person‐centredness change after implementation palliative care in nursing homes.

## 3 METHOD

This study is part of the project Knowledge‐based Palliative Care [in Swedish: KUnskapsbaserad PAlliativ vård”], abbreviated the KUPA project (Ahlström et al., 2018). Based on questionnaires and narrative descriptions collected by interviews with the older persons’ assessments, this study uses a mixed‐method design, suggested as a proper method for evaluating complex interventions (Creswell & Creswell, 2018; Farquhar, Ewing, & Booth, 2011). A convergent mixed‐method (Creswell & Creswell, 2018) was chosen since it can contribute to the development and evaluation of complex interventions and are particularly valuable in palliative care where interventions not seldom are complex and challenging (Farquhar et al., 2011). The purposes of the design are complementarity; that is, two methods (questionnaire and narrative answers) were used simultaneously to investigate the same phenomena in order to deepen and broaden the interpretations and conclusions.

### 3.1 Research setting

The evaluation of the KUPA project addresses several different outcomes reported in previous papers (Alftberg et al., 2018; Bökberg Behm, & Ahlström, 2019a, 2019b; Bökberg, Behm, Wallerstedt, & Ahlström, 2019; Bökberg et al., 2019) and will be further reported in future papers. The intervention was implemented during a 6‐month period with different staff and front leaders (8–12 in each nursing home) in 20 nursing homes in two different counties in southern Sweden (Ahlström et al., 2018). Trial registration: NCT02708498.

### 3.2 The Knowledge‐based Palliative Care Intervention

The Knowledge‐based palliative care intervention consisted of five 2‐hr seminars based on two Swedish national documents about principles of palliative care (National Board of Health and Welfare, 2013; Regional Co‐operative Cancer Centres, 2012), following the WHO definition of palliative care (Connor & Sepulveda Bermedo, 2014; Davies & Higginson, 2004; Hall et al., 2011. The focuses on the seminars were (a) palliative approach and dignified care; (b) next of kin; (c) existence and dying; (d) symptom relief; and (e) collaborative care. The content of the different themes had a common core, but space was left for discussions and questions connected to each of the themes. The seminar groups were led by experienced Registered Nurses and researchers from the field of palliative and geriatric care (Ahlström et al., 2018).

### 3.3 Sampling of nursing homes and participants

The selection of nursing homes was made through voluntary participation and resulted in larger and smaller nursing homes, from both urban and rural areas (Ahlström et al., 2018). The older persons were recruited consecutively in equal numbers from the implementation and the control nursing homes. In total, 90 older persons, (≥65 years) were included in the study based on the inclusion criteria of being Swedish speaking, not having dementia and with enough energy to manage a structured interview lasting up to one hour. Forty‐six dropouts were related to death, not enough energy and/or not interested in participating in the follow‐up interview. Altogether, 24 older persons in the implementation group and 20 in the control group were interviewed at both baseline and follow‐up (Table 1).

**Table 1:** Characteristics at baseline of the older persons in the nursing home intervention and control groups

Background variable	Intervention group (*N* = 24)	Control group (*N* = 20)
Age		
Year median (range)	87 (73–102)	88 (66–98)
Gender *N* (%)		
Women	16 (67)	11 (55)

### 3.4 Data collection

Since a convergent mixed‐method (Creswell & Creswell, 2018) was chosen, quantitative as well as qualitative data were collected with the same participants simultaneously within the same interview. Data were collected before the intervention started (baseline) and followed up three months after the intervention was completed (Ahlström et al., 2018). The included nursing homes were asked to designate a contact person who informed older persons, fulfilling the inclusion criteria, about the study. If the older person were interested in participating, the contact person informed the researchers about the older persons’ interest, and time and place for the interview were decided. With the intention of getting reliable data, the older persons answered the questionnaires in the form of a structured interview. Four interviewers, all Registered Nurses with experience in interviewing older persons presented the questions to the older persons. To make it easier for the older persons to answer, the response alternatives were enlarged on a separate paper. During the structured interviews, the older persons expressed their experiences of the care related to person‐centeredness. The interviews were recorded digitally and lasted on average 45–60 min.

### 3.5 Instruments

The data collection was based on two questionnaires, the Person‐centred Care Assessment Tool (P‐CAT) and the Person‐Centred Climate Questionnaire (PCQ). *The Person‐centred Care Assessment Tool (P‐CAT)* consists of 13 items that measure the extent to which care settings are rated as person‐centred. The instrument exists both in a staff version and in a version for older people. In this study, the version for older people was used. The instrument contains two subscales: (a) *Extent of personalization care*, containing eight items, and (b) *Amount of organizational and environmental support,* containing five items (Sjögren, Lindkvist, Sandman, Zingmark, & Edvardsson, 2012). The first subscale reflects intentions and actions of staff to prioritize personalized care and the possibility for residents to make own decisions. The second subscale includes factors in the environment and organization that can support or hinder the personalization of care. Informants are asked to respond on a 5‐point Likert scale, ranging from 1 (disagree completely)–5 (agree completely). The total score of the scale runs from 13–65, wherein higher values indicate a higher degree of person‐centeredness. In this sample, Cronbach´s alpha was α 0.80 for subscale 1 and α 0.75 for subscale 2.

*The Person‐centred Climate Questionnaire (PCQ)* consists of 17 items, to assess to what extent the climate of the care environment is person‐centred. PCQ exists both in a patient version (PCQ‐P) and in a staff version (PCQ‐S). In this study, the patient version was used and has proven to be valid and reliable (Edvardsson, Sandman, & Rasmussen, 2008). The subscales are (1) *Safety,* consisting of ten items; (2) *Everydayness,* consisting of four items; and (3) *Hospitality,* consisting of three items. Informants are asked to respond on a 6‐point Likert scale, ranging from 1 (No, I disagree completely)–6 (Yes, I agree completely). The total score of the scale runs from 17–102, wherein higher values indicate a higher degree of perceived person‐centeredness (Bergland, Kirkevold, & Edvardsson, 2012). In this sample, Cronbach´s alpha was α 0.86 for subscale 1, α 0.80 for subscale 2 and α 0.43 for subscale 3. One additional question was stated both at baseline and at follow‐up: “How do you rate your health?”, with possible responses ranging from 1 (very bad)–5 (very good) on a 5‐point Likert scale.

### 3.6 Analyses

#### 3.6.1 Statistical analysis

The quantitative data were analysed using methods applicable for within‐groups comparisons. The selection of methods, aside from descriptive statistics, was based on whether the data were distributed normally and the scale level of the instruments. The Wilcoxon signed‐rank test was used to compare paired data before and after the intervention. Subgroup analysis, using the Mann–Whitney U test, was applied to compare baseline characteristics of the intervention group and the control group. The same analyses were applied for the dropouts in the intervention group with the participants in the intervention group and the dropouts within the control group with the participants in the control group. Either the Pearson chi‐square test or Fisher´s exact test (if any expected value was <5) was used. Analyses were performed using IBM SPSS Statistics version 24. A two‐tailed *p*‐value of <.05 was regarded as statistically significant. Missing data on single items were replaced by the mean score for that item (Edvardsson, Fetherstonhaugh, Nay, & Gibson, 2010).

#### 3.6.2 Analysis of the transcribed interviews

During the structured interviews, 31 of the 44 older persons told the interviewee some personal spontaneous thoughts about their care and care environment. The text was subjected to qualitative content analysis (Graneheim & Lundman, 2004) performed by CB (Table 2). First, the verbatim transcripts were read through several times for overall understanding. Second, the older person's expressions were identified and divided into meaning units (sentences/paragraphs). Third, the meaning units were condensed at a descriptive level, keeping close to the text. Fourth, the condensed meaning units were abstracted and labelled with a code. The interviews in their entirety served as a point of reference throughout the analytical process, particularly when deeper understanding was needed with respect to the meaning units and codes. The codes were thoroughly compared to check for similarities, and differences before categories were created. Finally, the author (GA) read the transcripts and reviewed the content carefully in relation to the meaning of each category.

**Table 2:** Example of analysis steps

Meaning unit	Condensed meaning unit Description close to the text	Code	Category
Yes, it could be more (laughs) but they are doing the best they can, they need to hand out food to everybody before they can sit down and talk to us. It must feel very stressful for them as well. They have to do that first, there need to be more staff and that's all understood. That's the way society is today, but I'm not complaining. And they are so nice and so kind. But I do not want more service than I need (laughter)	…they are doing their best, they need to share the food to everybody before they can sit down and talk to us. It must be stressful for them as well… there need to be more staff… but I am not complaining…	Putting the own needs behind Understanding of staff situation	Routines first then the older person
…they are too busy with the others… I don´t think that the health care would have been enough… there are need for big changes, very big… if I didn´t have my children… they are such a big help… they come and walk with me, we go shopping… it would have been much more boring without them… it is very few days they don´t visit… but I can´t expect them to come every day and activate me… nurses they are so occupied, but the children they concentrate	… I don´t think that the health care would have been enough… if I didn´t have my children… they are such a big help… nurses they are so occupied, but the children they concentrate	Shortcomings of the staff Responsibilities on next of kin	Putting demands on next of kin

#### 3.6.3 Mixed‐method analysis

After the both data sets have been analysed separately, the process of integration the findings from the two different methods took place, that is the interpretation stage of the mixed‐methods analysis. The categories from the quality analysis were jointly displays (Fetters, Curry, & Creswell, 2013) under each subscale of the instruments. The findings from each data set were reflected on by all in the research team if the data sets agreed, offer complementary information, or contradict each other.

#### 3.6.4 Ethical considerations

The KUPA project was approved by the Regional Ethics Review Board, Lund (reference number: 2015/4), and guided by research ethical principles for medical research in accordance with the ethical standards of the Declaration of Helsinki (World Medical Association, 2013). The participants were given verbal and written information about the study before the verbal informed consent and the written consent were signed. The participation was voluntary and could be interrupted at any time without having to give a reason and without any consequences.

## 4 RESULTS

No statistically significant differences were detected when comparing baseline characteristics of the two groups. This was the same result when comparing baseline characteristics and the subscales between the dropouts in the intervention group with the participants in the intervention group and the dropouts within the control group with the participants in the control group.

The mixed‐method analysis showed that the quantitative and qualitative results were complementarity to each other. Therefore, all results are presented according to the categories of the subscale names jointly displayed (Table 3).

**Table 3:** Scales, subscales and categories

Scales and subscales	Categories
P‐CAT	
Extent of personalizing care	Routines first then the older person
Amount of organizational and environmental support	Putting demands on next of kin
PCQ‐P	
Safety	Not feeling safe related to shortcomings in the staff
Everydayness	Being able to take part in everyday activities and social interaction
Hospitality	The importance of a familiar environment

### 4.1 Person‐centred care

The overall results of the P‐CAT showed no statistically significant changes concerning person‐centeredness after implementation of the KUPA intervention for either of the two subscales: *Extent of personalizing care* and *Amount of organizational and environmental support* (Table 4).

**Table 4:** Intervention group (*N* = 24) and control group (*N* = 20) analyses before and after implementation

Scale and subscale	Intervention group Baseline Median (Q1‐Q3)	Intervention group Follow‐up Median (Q1‐Q3)	Changes after compared with before p‐value^a^	Control group Baseline Median (Q1‐Q3)	Control group Follow‐up Median (Q1‐Q3)	Changes after compared with before p‐value^a^
P‐CAT						
Extent of personalizing care (8−40 b)	24. 5 (17.5–30.8)	25.0 (18.0–32.0)	0.659	29.0 (22.0–31.0)	27.0 (22.2–29.8)	0.545
Amount of organizational and environmental support (5−25 b)	11.0 (6.5–16.5)	10.0 (6.5–15.8)	0.887	12.0 (7.2–15.0)	11.0 (10.0–16.0)	0.447
PCQ‐P						
Safety (10−60 b)	55.0 (50.0–57.2)	51.5 (48.5–55.8)	**0.018**	56.0 (48.5–59.8)	53.0 (47.5–58.0)	0.878
Everydayness (4−24 b)	19.5 (17.0–23.0)	20.0 (18.0–23.0)	0.563	20.5 (18.0–23.0)	19.5 (18.0–22.0)	0.630
Hospitality (3−18 b)	15.0 (14.0–16.0)	16.0 (13.2–18.0)	0.368	16.0 (13.5–18.0)	15.5 (14.2–17.0)	0.959

Note

Q1 = first quartile; Q3 = third quartile. Significant values are given in bold.

a Wilcoxon signed‐rank test.

b Underlined score is the most favourable score.

### 4.2 Extent of personalizing care – Routines first then the older person

The median score for the subscale *Extent of personalizing care* was rated as median score 24.5 before the intervention and as 25.0 after the intervention, *p*‐value .659 (Table 4).

The older persons’ experiences expressed in the category *Routines first then the older person*, and they narrated that the daily routines, such as cleaning and making beds, were much more important for the staff than taking time to sit down to talk to them, creating meaningful activities or making a homelike environment. To find time to just sit down and talk seemed like impossible from the older persons’ perspective, instead the staff combined daily routines and talking. More staff and more time were requested by the older persons as they found that the staff were stressed trying to handle all the routines, making it impossible for the older persons to find the time or right moment to express needs or just to have a chat. Likewise, time for discussions concerning care planning and visiting outdoor activities or leisure activities was sparse. Despite these shortcomings, the older persons expressed lots of understanding for the staff´s situation, putting themselves behind, not complaining and trying to manage on their own:

*…they have so much to do… it is rather difficult to reach them… they are not more than two at each ward… they are cleaning, making the beds… I mean they must do that ‐ if it not that anyone is dying* (man 92 years, control group)

*…they are doing their best, they need to share the food to everybody before they can sit down and talk to us. It must be stressful for them as well… there need to be more staff… but I am not complaining…* (woman, 87 years, control group)

### 4.3 Amount of organizational and environmental support – Putting demands on next of kin

For the subscale *Amount of organizational and environmental support,* the median score was 11.0 before and 10.0 after the intervention (*p* = .887), see Table 4.

The obstacles experienced by the older persons for staff with respect to talking and spending time with them are also relevant to the content of the category *Putting demands on next of kin*. Lack of time and staff as well as the shortcomings of the staff meant that next of kin needed to take on additional responsibilities, as described by the older persons. These responsibilities concerned everyday activities and little things to improve the older persons´ quality of life. However, the older persons seemed to be worried about putting demands on their next of kin, but the situation forced them to do so:

*…if I haven´t got my daughter, coming and take me out for a walk and shopping, then it would be much more boring… but I can´t expect her to be here all the time* (woman, 87 years, control group)

*… if I didn´t have my children… they are such a big help… nurses they are so occupied, but the children they concentrate* (woman, 98 years, implementation group)

### 4.4 Person‐centred Climate

The instrument PCQ‐P measured changes in the climate concerning person‐centeredness after the implementation. The results for the intervention group revealed a statistically significant decline on the first subscale *Safety,* but not in any of the items of this subscale. No statistically significant changes in the control group were detected for any of the three subscales (Table 4).

### 4.5 Safety – Not feeling safe related to shortcomings in the staff

The older persons rated their feelings of safety before the intervention at a median score of 55.0 and after the intervention as 51.5 (*p* = .018), see Table 4. The results from the qualitative data showed that taking care of older persons requires specific competencies and education expressed in the category *Not feeling safe related to shortcomings in the staff.* However, the older persons stated that these specific competencies and education often were lacking. Furthermore, the older persons requested older staff with life experience and common sense as well as regular staff versus temporarily employed staff. During holidays, when the ordinary staff were on vacation, the routines failed, and the older persons felt that the temporary staff did not know them or their needs and preferences. These failures resulted in feelings of insecurity and that their needs and preferences were not being fulfilled. Also, the ability contacting staff with higher competences, such as a Registered Nurse or general practitioner, was not an easy task. Other problems were related to staffs´ shortcomings in communication, when the older person and the staff did not speak the same language or when the staff spoke too quickly or too low. These shortcomings resulted in feelings of insecurity and a lack of safety in the older persons:

*…this summer, it has been a lot of changes… there are new staff everyday… it become messy… the best is when the ones that use to be here are coming because they know how we use to have it…* (woman, 88 years, implementation group)

*…they left me in the evening at the toilet… and there I sat for half an hour, alarming, and alarming… nobody come and helped me* (woman, 87 years, control group)

*… no one is coming in on the evening to say good night … I can fall into the floor, I can even lie and die. How long shall I be laying before they discover when they don´t come in on the evening* (woman, 88 years, implementation group)

### 4.6 Everydayness – Being able to take part in meaningful activities and social interaction

For the second subscale: *Everydayness,* the median score was before the intervention 19.5 and after 20.0, *p*‐value = .563 (Table 4). The older persons expressed *being able to take part in meaningful activities and social interaction*, as meaning having the opportunity to take part in different types of activities they perceived as meaningful, such as entertainment (bingo, dancing, singing), church activities, physical activities (walking, gymnastics, bowling), senior meetings, reading and lessons. Being able to partake in everyday activities, such as cooking and cleaning, was also described as meaningful, pleasant and welcoming elements in their daily lives. Furthermore, being able to partake in social interaction was meaningful for the older persons. This meant discussing common persons that they have known during their lifespan and being recognized as someone other than “an older person at a nursing home.” The older persons expressed that social interaction with the staff was facilitated by humour, joking and a happy mood. However, the older persons described difficulties in taking part in meaningful activities due to their own energy level and difficulties in sight and hearing. These difficulties, along with cognitive impairments and advanced diseases, made it difficult to interact with other residents. The older persons also described the loss of old friends and difficulties in getting new ones as barriers for social interaction:

*…you can´t judge everyone that lives here, but some of them are rather senile, and it can be tedious when you ask and get the same answer ten times…* (man, 86 years, control group)

*…best of all is that they* [the staff] *are so cheerful and that they can make joke to you, that I think is great… it cheers up* (woman, 80 years, control group)

### 4.7 Hospitality – The importance of a familiar environment

The results for the third subscale *Hospitality* were 15.0 before the intervention and 16.0 after, *p*‐value = .368 (Table 4). The qualitative analysis revealed that both the internal and the external environments were reflected on by the older persons. The category *the importance of a familiar environment* showed that to be able to remain living in the same village as the older persons were used to was important for their well‐being. This arrangement made it possible for the older persons to make excursions into the village and visit familiar places. For those older persons who lacked the energy to go outside, they described the importance of being able to look out the windows and see the season changes, which was facilitated by plants near the nursing home. Some of the older persons said that they had the opportunity to go outside and that the staff willingly helped them. The older persons also reflected that when building nursing homes, it is important that it looks like a home and not a hospital:

*We have a nice environment here; we go out and they accompany me to the shop and the church and my parents’ graveyard. Yes, I am so pleased to be able to live here where I have grown up…* (man, 88 years, control group)

*You don´t build nursing homes that look like corridors and hospitals… It doesn't create the community as it could have been* (man, 85 years, implementation group)

The self‐rated health status in the intervention group was unchanged compared with baseline (median score 3.0, q1–q3 = 2.0–4.0, *p*‐value = .508), but in the control group it revealed a non‐significant trend of declined health status from the baseline median score 3.0 (q1–q3 = 3.0–4.0 to median score 3.0 (q1–q3 = 3.0–3.0) at follow‐up, *p*‐value = .097).

## 5 DISCUSSION

The only significant change was that safety decreased, also expressed by the older persons as not feeling safe. The spontaneous narratives of the older persons list shortcommings in staff such as no education, no previous experience of caregiving and not being able to speak Swedish as reasons for this. This is confirmed by a Swedish study of staffs’ perceptions of person‐centred care in nursing homes (Sjögren, Lindkvist, Sandman, Zingmark, & Edvardsson, 2015) which found that lower levels of job strain, high levels of conscious stress, higher levels of staff satisfaction and supportive psychosocial climate for the staff were associated with person‐centred care. A reasonable explanation of the decrease in safety in the intervention group can therefore be shortage of staff leading to a high workload, high staff turnover and/or an insufficient leadership.

The analysis of the transcribed interviews gives a picture of how the older persons experienced their care in the nursing homes in spontaneous comments in connection with the structured interviews. They reflect about both negative and positive aspects of their care and the care environment. One of the experiences was that the staff were concentrated on the practical tasks rather than being available to socialize with the older persons. This fact needs attention as social relationships have been identified as essential to nursing home residents’ quality of life (Bergland & Kirkevold, 2005; Custers, Westerhof, Kuin, Gerritsen, & Riksen‐Walraven, 2012). Consistent with our results, earlier research has shown that the majority of staff–resident interactions are task focused (Bowers, Esmond, & Jacobson, 2000; Bowers, Fibich, & Jacobson, 2001; Bowers, Lauring, & Jacobson, 2001). These missed opportunities for interactions with the residents reduce the staff's opportunities to get to know residents on a personal level (in‐depth understanding of the older person's preferences, norms and needs; Bowers et al., 2000) and thereby hampers person‐centred care. The older persons in our study also stated that the staff did not put much time in making the environment homelike. Making the environment homelike is an important aspect of care since previous studies have shown that a homelike environment is important for older persons to feel satisfied with the care at nursing homes (Murphy, 2007; Rasmussen & Edvardsson, 2007).

### 5.1 Methodological limitations

This study was designed as a part of an evaluation of a complex intervention (Ahlström et al., 2018), characterized by several interacting components, with a range of possible outcomes, or variability. This complexity threatens construct validity since the effects are difficult to attribute to certain components or specific “active ingredients.” Therefore, the development and feasibility of the intervention were carefully described in a previous study (Ahlström et al., 2018). One way to increase the validity of the result about person‐centred care and to understand whether and how the intervention work or does not work is to use a mixed‐method design to evaluate the effectiveness of the intervention (Farquhar et al., 2011). In this study, we explore the knowledge value of spontaneous narratives that emerged when asking older persons about person‐centred care and climate. More comprehensive interviews could have led to deeper statements. However, for research ethical reasons it was of importance protecting the older persons from exhaustion caused by unnecessary data collection. The interviewers were skilled nurses who split interviews over two sessions when it was necessary for protecting the older persons from too much pressure. This sensitivities and empathy are required from palliative care researchers (Farquhar et al., 2011). In addition, the digital recording was used under the entire interview in order to avoid switching on and off the recorder which meant that the interviewer was able to have the whole attention on the older person, not the recorder equipment (Farquhar et al., 2011). In future research including frail older persons, we recommend shorter interviews to receive a higher quality of the data due to the difficulty for older persons to endure longer interview situations.

To carry out complex interventions including frail older persons with palliative care needs involves several methodological challenges (Farquhar et al., 2011). We would like to discuss some of them. Frail older persons are commonly excluded from research due to their vulnerable position (Smedbäck et al., 2017; Ternestedt & Franklin, 2006; Towsley, Hirschman, & Madden, 2015). However, as the proportion of frail older people increases, the importance of developing research‐based knowledge about good care increases. The challenge of recruiting older persons and retaining them over time needs to be considered. In this study, we first encountered problems finding older persons that were mentally and physically able to answer questions reliable in a 1‐hr interview. Most of them had some kind of cognitive impairment, and most of them who did not have impairments were very frail. This is consistent with previous research (Björk, et al., 2016; Hoffmann, Kaduszkiewicz, Glaeske, van den Bussche, & Koller, 2014), concluding that most persons living in nursing homes have some kind of cognitive impairment. We attempted to include at least 90 persons, which we did; however, the dropout rate was considerable (51%) and higher than expected, leaving us with a limited number of persons (*N* = 44) included in the analytical part, decreasing the generalization of the results. Even though there were no including criteria regarding need for palliative care, it could be supposed that all older persons living in nursing homes need palliative care, since the length of stay at nursing homes is decreasing (Schon et al., 2016; Smedbäck et al., 2017). The lesson learned is to include a larger number of nursing homes to reach enough statistical power.

The follow‐up period was rather long, nine months, which is also the average length of stay at nursing homes in Sweden (Schon et al., 2016). Thus, when conducting experimental studies including older persons in the future, a narrower follow‐up period is recommended. However, a shorter follow‐up period means a shorter intervention, which, in turn, threatens the effectiveness of the intervention.

The patient version of the P‐CAT had not been tested before and is not validated. The questionnaire seems easy to complete with few questions and could therefore be considered suitable for older persons. However, we found that the older persons had difficulties understanding the response alternative (five or six), which resulted in some missing answers. Some questions in the P‐CAT and PCQ‐P had an inverse statement, or were similar to each other, which were confusing for the older persons. One action that we took to make the response alternatives easier to understand was to enlarge the answers. However, we had to ask the questions several times, but the shift between the question and response alternative was still difficult for the older persons. Despite the difficulties in collecting data, the reliability test revealed a result within the recommended values, except for the subscale *Hospitality* (Cronbach *α* 0.43). This subscale consists of only three items and need to be considered when interpreting the results. According to Doll, Cornelison, Rath, and Syme (2017), it is difficult and takes a long time to change a care culture. We measured person‐centred care and care climate three months after the intervention was completed, which might have been too short time period to detect a change. Also, only 8–12 staff at each nursing home were involved in the intervention, which might have been too few to detect a change.

## 6 CONCLUSIONS

This study contributes valuable knowledge about evaluation methods that need to be considered when measuring the outcomes of interventions in nursing homes. The results indicated that the dose of the intervention was not sufficient to improve person‐centred care, given that only 8–12 staff at each nursing home were involved in the intervention. Future research could preferably include and educate all staff before measuring if any improvements occurred.

The outcomes measured were from the perspective of frail older persons, which often are excluded from research. However, the dropout rate related to death and not enough energy was considerably high even though one of the inclusion criteria was to have enough energy to manage a 1‐hr interview. This result supports previous research describing the difficulties in recruiting and retaining frail older persons in research.

## CONFLICT OF INTEREST

The authors have no conflict of interest to declare.

## AUTHORS' CONTRIBUTIONS

CB: Quantitative and qualitative analysis and drafting the article. GA (project leader): development of the design; the recipient (PI) of the national research grant, monitoring the recruitment, analysis and critical revision of the results. BW and LB: Interview of the older persons. All authors contributed to the content of the manuscript text, critically reviewed, discussed, and approved the final manuscript.
